# ADTC-InSAR: a tropospheric correction database for Andean volcanoes

**DOI:** 10.1038/s41597-022-01630-w

**Published:** 2022-08-27

**Authors:** Fernanda Lopez-Pozo, Rodrigo Abarca-del-Rio, Luis E. Lara

**Affiliations:** 1 Doctorado en Ciencias Geológicas, Departamento de Ciencias de la Tierra, Facultad de Ciencias Químicas, Universidad de Concepción, Concepción, Chile; 2 Departamento de Geofísica (DGEO), Universidad de Concepción (UDEC), 160-C Concepción, Chile; 3 Servicio Nacional de Geología y Minería (SERNAGEOMIN), Avda. Santa María, 0104 Santiago, Chile; 4 Centro de Investigación para la Gestión Integrada del Riesgo de Desastres (CIGIDEN), Av. Vicuña Mackenna 4860, Macul, Santiago, Chile

**Keywords:** Volcanology, Geophysics, Natural hazards

## Abstract

Monitoring geophysical hazards requires a near real-time response and precise interpretation of InSAR data, typically recording minute surface deformations. Accurate tropospheric adjustment is an essential aspect of InSAR processing. This study provides a free database of ready-to-use Tropospheric Correction for InSAR for the three volcanic zones from north to south of the Andes. Average Daily Tropospheric Correction for InSAR (ADTC-InSAR) is a collection of average daily tropospheric delay matrices created using ECMWF re-analysis of the global atmosphere and surface conditions (ERA5) as atmospheric data and TRAIN software. The construction method and annual variation according to the climatic zones are provided, and its effectiveness is evaluated. ADTC-InSAR facilitates the generation of tropospheric corrections in InSAR with easy access, fast application, and accuracy comparable to TRAIN. Its purpose is to serve as a starting point for tropospheric correction in the event of emergency response to extreme occurrences and as a reference for other research and academic objectives.

## Abstract (summary)

**Table:** 

Measurement(s)	Average daily tropospheric delay
Technology Type(s)	Matlab m file
Factor Type(s)	atmospheric pressure • atmospheric temperature • relative humidity • Digital Elevation Model
Sample Characteristic - Environment	area of barren land
Sample Characteristic - Location	Andean Region

## Background & Summary

Interferometry Synthetic Aperture Radar (InSAR), a technique based on a phase difference between two images, allows the study of Earth’s ground deformation induced by earthquakes^1^, mass wasting in landslides^2,3^, volcanic unrest, and deflation after magma withdrawal^4–6^, among the others. Over the past decade, the introduction of new constellations of satellites with higher capacity and more advanced sensors resulted in an unprecedented ability to track the temporal evolution of changes across the entire Earth’s surface. This implies an increasing number of scenes in accessible databases and the need for automated analysis, which is an effort that the scientific community is releasing^7,8^.

A source of error of InSAR is the atmospheric delay caused by the refraction of the signal as it crosses the troposphere, resulting in a change in trajectory. The tropospheric delay has a dry component (or dry contribution) determined by atmospheric pressure and temperature. The order of magnitude is in meters and is affected by topography. The wet component (or wet contribution) is a function of the partial pressure of water vapor. As a result, it is influenced by the turbulent region of the atmosphere and is not affected by topography; it has a centimeter order of magnitude.

The importance of the tropospheric delay lies in the magnitude of the changes generated, considering that volcanic areas usually show ground deformations in the order of a few centimeters. Zebker *et al*.^9^ indicates that a change of 20% in relative humidity can generate a difference between 10 and 14 [cm] of delay, which can be more significant than the detected deformation even considering that the wet component only represents 10% of the total^10^. A more precise measurement of the tropospheric delay throughout the year is required for the most accurate interpretation. We can see this at Llaima volcano (Southern Andes, Chile: [−71.730°, −38.697°]) where different interpretations have been generated from InSAR. According to Fournier *et al*.^11^, between 2007–2008, there was subsidence on the volcano’s eastern flank, which is presumed to be related to the January 2008 eruption and collapse. Bathke *et al*.^12^ specify that a deflation of: 10 [cm] occurred between 2003 and 2007, followed by: 8 [cm] of inflation that lasted until the end of 2008. Delgado *et al*.^13^ interpreted the interferogram signal one month before the April 2009 eruption as: 6–15 [cm] inflation of the west side of the volcanic edifice. Remy *et al*.^14^ stated that no deformation was evidenced between 2003–2011 and that the patterns observed in the interferograms are the product of tropospheric error but pointed out the collapse of the eastern flank in 2008.

The Andes is a natural laboratory for InSAR studies due to the variety of active processes and latitudinal and topographic variation. This offers diverse climatic conditions in which the humid input would exert significant and different control on the tropospheric delay in each zone.

Methods for determining this delay range from using correlation with topography to more sophisticated models. Regardless of quality, heterogeneity in the treatment of this aspect hinders remote sensing studies from being compatible and comparable.

**Fig. 1:**
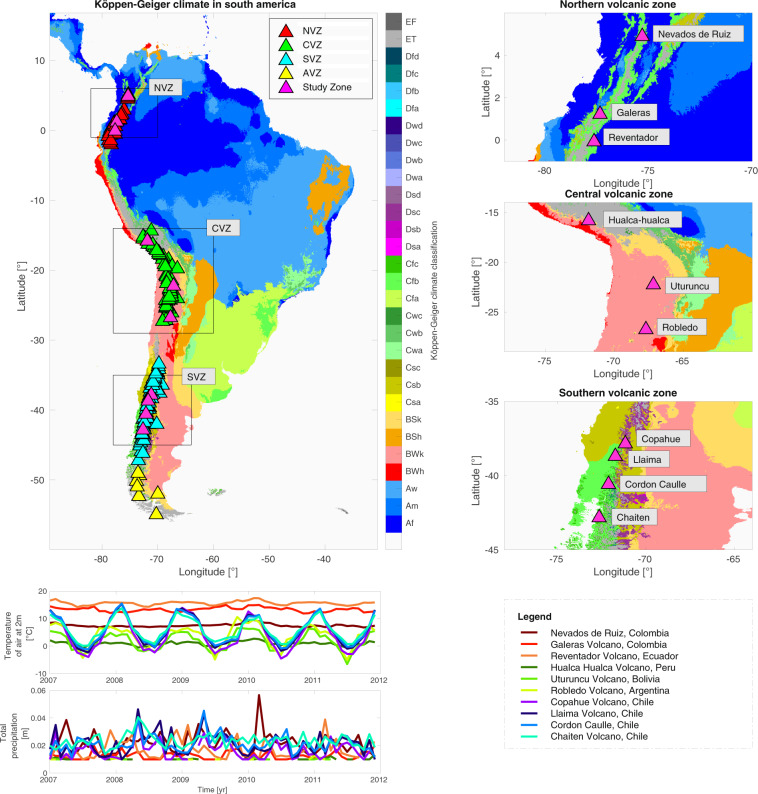
On the upper left of the figure are the Koppen-Geiger Climates^15^, and the color bar on the right represents each Koppen-Geiger climate. The Andes Mountains volcanoes are divided into three zones: North Volcanic Zone (NVZ), the Central Volcanic Zone (CVZ), and Southern Volcanic Zone (SVZ). We extracted from this maps, the study areas shown on the right, which comprise the following volcanoes (magenta triangles): Nevados del Ruiz, Galeras, Reventador, Hualca-hualca, Uturuncu, Robledo, Copahue, Llaima, Cordón Caulle, and Chaitén. The two graphs on the bottom left offer time series of precipitation and air temperature at 2 meters for each volcano, with the legend on the right. Note that each volcanic zone is distinguished by a distinct color range: the Northern Volcanic Zone is red, the Central Volcanic Zone is green, and the Southern Volcanic Zone is blue. Note that Austral Volcanic Zone (AVZ) in yellow are not analyzed.

We aimed to investigate this topic through approaches based on globally available and recognized atmospheric data. As a result of this study, Average Daily Tropospheric Correction for InSAR (ADTC-InSAR) database has been developed using ERA-5 atmospheric reanalysis to produce a homogeneous and high-quality correction that offers information on tropospheric delay behavior at the continental scale. It is provided for three significant volcanic segments in the Andes that exhibit a range of Koppen-Geiger climates^15^. These are the Northern Volcanic Zone (NVZ), the Central Volcanic Zone (CVZ), and the Southern Volcanic Zone (SVZ). These zones have tropical, desert, and temperate climates, respectively (see Fig. 1).

We propose an ADTC-InSAR database that may assist in the research and understanding of tropospheric delay magnitudes and to understand better how the wet and dry components of the delay fluctuate in response to the prevailing climate. With this database, we were able to generate tropospheric corrections with good precision and fast application for InSAR data in the volcanic areas used as a case study.

## Methods

Historically, the tropospheric delay needed for interferogram correction has been measured with a number of methods. These methods must consider complementary data in order to generate a tropospheric correction^16^. Some methods generate corrections considering the correlation with topography^4,17,18^, time and/or spatial filters^19–25^, InSAR or GPS time series^26–30^, using methods that involve the reconstruction of the path of the ray and estimation of the distance traveled^31–34^, and obtaining the tropospheric delay of the signal utilizing the integral of the refractivity through the atmosphere by using atmospheric variables such as the total tropospheric pressure, the temperature, and the partial pressure of water vapor^14,21,35–43^. This is beneficial when the connection between delay and elevation is not linear^44^. As will be detailed later, we will utilize a program that implements the latter strategy, which requires atmospheric data.

The study area consist of the 10 volcanoes of the Andean volcanic arc: Nevados Del Ruiz, Galeras, and Reventador in NVZ; Hualca hualca, Uturuncu and Robledo in CVZ; Copahue, Llaima, Cordón Caulle and Chaitén in SVZ. A distinct prevailing climate surrounds each volcanic zone. We identify mainly tropical, desert, and temperate climates in the NVZ, CVZ, and SVZ zones, respectively^15^ (see Fig. 1).

### Generation of interferograms

For the generation of interferograms, we use Generic Mapping Tools Synthetic Aperture Radar (GMTSAR) software^45,46^ (https://topex.ucsd.edu/gmtsar/) with Land Observation System Phased-Array Synthetic-Aperture Radar (ALOS-PALSAR) and Shuttle Radar Topography Mission 3 arc-Second Global Digital Elevation Model (DEM-SRTM^47^; https://www2.jpl.nasa.gov/srtm/) data. GMTSAR utilizes the p2p_ALOS.csh code to generate the interferograms with ALOS-PALSAR data. This function does data preprocessing, focuses and aligns Single Look Complex (SLC) images, filters the interferogram, unwraps phase, and geocodes the data.

For running GMTSAR, the configuration file config.ALOS.txt is required. In this file, these options have been selected: *topo_phase*=1, for topographic correction; *switch_master*=0, to utilize the master image as a reference; *filter_wavelength*=300, for the interferogram filter; *correct_iono*=0, to avoid generating ionospheric corrections; and *threshold_snaphu*=0.1, for phase unwinding with snaphu.

Advanced ALOS PALSAR (https://asf.alaska.edu/data-sets/sar-data-sets/alos-palsar/) data from the Japanese Aerospace Exploration Agency (JAXA, https://global.jaxa.jp/) between 2007 and 2011 were utilized to generate interferograms (see Table 1). The advantage is due to its L wavelength (1.27 GHz), which permits higher penetration into the vegetation cover and lesser correlation loss. The utilized data contain Fine Beam Single (FBS) and Fine Beam Double (FBD) HH + HV polarization (where H: horizontal and V: vertical), with 20 and 10 m spatial resolution and an incidence angle of 34.3°; they were downloaded from https://search.asf.alaska.edu/#/. In addition, topographic information is required for topographic and tropospheric corrections. DEM-SRTM3^47^, measured vertically in meters and with a 90 m spatial resolution (downloaded from https://topex.ucsd.edu/gmtsar/demgen/) has been utilized.

**Table 1:** Table showing the SAR images used to generate interferograms in each of the volcanoes (column 1) with their respective abbreviations (column 2) in the study area (section 2) including the path (column 3) corresponding to ALOS-PALSAR images, the date (column 4 and 7) on which the image was captured (MASTER and SLAVE), its absolute orbit (column 5 and 8) and acquisition mode (FBS: Fine Beam Single Polarisation; FBD: Fine Beam Dual Polarisation; column 6 and 9) are showed.

Volcano	Abbr.	Path	Master			Slave			Baseline	
			21.5 cm Date (yyyymmdd)	21.2 cm Absolute Orbit	20.9 cm Beam Mode	21.5 cm Date (yyyymmdd)	21.2 cm Absolute Orbit	20.9 cm Beam Mode	21.3 cm Temporal [Days]	21.2 cm Spatial [m]
Nevados Del Ruiz	NDR	447	20080306	11269	FBS	20100312	22005	FBS	736	187.73
Galeras	Gal	152	20070303	5880	FBS	20080905	13932	FBD	552	−319.262
Reventador	Rev	108	20080107	10401	FBS	20090224	16440	FBS	418	148.619
Hualca-hualca	HHu	103	20100119	21239	FBS	20110122	26607	FBS	368	−1236.799
Uturuncu	Utu	98	20090320	16644	FBS	20100313	22012	FBS	358	−1219.686
Robledo	Rob	101	20100318	22085	FBS	20101219	26111	FBS	276	−227.810
Copahue	Cop	115	20101227	26228	FBS	20110211	26899	FBS	46	228.07
Llaima	Lla	116	20071120	9701	FBS	20071005	9030	FBD	46	−201.040
Cordón Caulle	CCa	118	20100213	21604	FBS	20100331	22275	FBS	46	−734.158
Chaitén	Cha	121	20100218	21677	FBS	20070928	8928	FBD	874	6.487

The temporal baseline (column 10) and spatial baseline (column 11) are also displayed.

### Generation of ADTC-InSAR

The average tropospheric correction was constructed using TRAIN (Toolbox for Reducing Atmospheric InSAR Noise^42,43^; https://github.com/dbekaert/TRAIN) with ERA-5 atmospheric^48,49^ and DEM SRTM3^47^ data.

The tropospheric correction is based on the ERA-5 atmospheric reanalysis that superseded ERA-Interim. Thus, whereas ERA-Interim had an 80 km horizontal spatial resolution and 60 vertical levels to 0.1 hPa, ERA-5 now has a 30 km horizontal spatial resolution and 137 vertical levels from the surface to above 80 km altitude https://www.ecmwf.int/en/forecasts/datasets/reanalysis-datasets/era5). Additionally, the temporal resolution also increased from 6 hours to 1 hour. The European Centre for Medium-Range Weather Forecasts provides the ERA-5 data (ECMWF; https://www.ecmwf.int/) via its ECMWFAPI library https://pypi.org/project/ecmwf-api-client/).

For each day between 2007 and 2011, the dry, wet, and total daily tropospheric delay were calculated using TRAIN to build the daily average database. Then, the daily tropospheric delays for each day between 2007 and 2011 are then used to build a daily average grid for a whole year. For example, the matrices produced on January 1, 2007, 2008, 2009, 2010, and 2011 are averaged to obtain a single mesh. This term refers to the average daily tropospheric correction for dry, wet, and total conditions.

### Tropospheric correction

To compute the tropospheric correction using the daily average database, it is necessary to recollect the dates when the SAR images for the interferograms were acquired: date_MASTER and date_SLAVE. We require the ADTC-InSAR of the total daily tropospheric delay corresponding to the previously indicated dates. If date_MASTER is August 6, 2009, we search for August 6 in the ADTC-InSAR. This results in two meshes representing the ADTC-InSAR of the daily total tropospheric delay for the dates date_MASTER and date_SLAVE: mesh_MASTER and Mesh_SLAVE. The meshes are interpolated so that their points match those in the unwrapped interferogram. The data is converted from centimeters to radians, and the difference between their points is computed: mesh_SLAVE-mesh_MASTER. Thus, ADTC-InSAR, has made the tropospheric correction for the interferogram under consideration available.

### Analysis of average daily tropospheric delay

Two lines of analysis have been developed for evaluating the information of the daily average tropospheric delay: (1) to investigate the temporal behavior at the seasonal and annual time scales using various statistics such as median, mean, and standard deviation; and (2) to investigate the relationship between the total daily tropospheric delay, dry and wet contribution with Koppen-Geiger climates. As will be detailed further, the northern, central, and southern volcanic zones are characterized by their tropical, desert, and temperate climates, respectively.

### Analysis of tropospheric correction with ADTC-InSAR

This work aims to examine the efficiency of using ADTC-InSAR as a tropospheric correction. This consists of knowing if ADTC-InSAR is appropriate for use as a generic correction prior to, as a first guess, or even in place of data from specific dates, which is the common practice. For doing this, we compare a correction generated by ADTC-InSAR to one generated with specific dates. It is worth noting that specific dates cover days found throughout the 5 years window (2007–2011), while ADTC-InSAR only queries the day and month considered.

Thus, for each volcano, we generated two databases of tropospheric corrections for 500 random date pairings (described further below) to be compared, which we refer to as corrections with “ADTC-InSAR” and “TRAIN specific-dates”.

ADTC-InSAR correction implies locating the matching daily dates in the ADTC-InSAR database generated for the volcano to acquire the corresponding tropospheric correction. In contrast, “TRAIN specific-dates” tropospheric correction entails producing the tropospheric corrections for InSAR conventionally, i.e., using TRAIN, for each previously selected random date. We intend to determine the degree of similarity and difference by comparing the magnitudes of the results produced by each method in each volcano.

It is worth mentioning that a Monte-Carlo process is applied to choose these 500 pairings of random dates. That is a well-recognized approach for tackling estimation and optimization problems^50^ in a wide variety of domains, including statistics, mathematics, and the physical sciences. However, the statistics barely vary significantly more than 100 dates after the volcano testing, and at 300, they level off. Consequently, 500 corrections were made with “TRAIN specific-dates” data (from 2007 to 2011) and 500 with ADTC-InSAR. The difference between identical pairs of day corrections was then calculated.

In this section, different statistics are applied depending on the instance. In the first place, the mean (*μ*, Eq. 1), median, standard deviation (*σ*, Eq. 2), quartiles 1 and 3, kurtosis (*k*, Eq. 3), and skewness (*s*, Eq. 4) are used to appreciate the data distribution of the corrections for 500 pairs of random dates in each volcano and the difference between the corrections. For example, the mean, median, standard deviation, and quartiles allow us to understand where the data are concentrated. At the same time, kurtosis and skewness provide information on how they are distributed.

The statistics used are defined in the following equations:

1 $$\mu =\frac{1}{n}\mathop{\sum }\limits_{i=1}^{n}{x}_{i}$$

2 $$\sigma =\sqrt{\frac{1}{n-1}\mathop{\sum }\limits_{i=1}^{n}{\left|{x}_{i}-\mu \right|}^{2}}$$

3 $$k=\frac{E{\left(x-\mu \right)}^{4}}{{\sigma }^{4}}$$

4 $$s=\frac{E{\left(x-\mu \right)}^{3}}{{\sigma }^{3}}$$

Where, *x*_*i*_ is the ith observations, *μ* is the mean, *σ* is the standard deviation, *n* is the number of the observations, and *E(x)* represents the expected value of the quantity *x*. Moreover, quartiles 1, 2 and 3 (*Q*_1_, *Q*_2_ and *Q*_3_) correspond to measures of data distribution, which has scalar values. *Q*_1_, *Q*_2_ and *Q*_3_ correspond to the 25th, 50th and 75th percentiles of the data distribution. In particular, *Q*_2_ is equivalent to the median.

In second place, when comparing the corrected interferograms with “TRAIN specific-dates” and those with ADTC-InSAR, the squared correlation coefficient (“R squared”, *R*^2^, Eq. 5), Nash-Sutcliffe coefficient^51^ (*nse*, Eq. 6), and the modified Willmott coefficient^52^ (*d*, Eq. 7) are utilized (in Table 4). These statistics allow two data sets to be compared to estimate their similarity. The “R squared”, or the coefficient of determination provides information on how well the “ADTC-InSAR” approximates the “TRAIN specific-dates”, when *R*^2^ = 1, these data sets fit perfectly. The Nash-Sutcliffe coefficient varies between inf and 1; when *nse* = 1, the data sets match perfectly. Furthermore, finally, the Willmott coefficient varies between 0 and 1; when *d* = 1, the data have a perfect agreement, and if *d* = 0, there is no agreement.

The statistics used are defined in the following equations:

5 $${R}^{2}={\left[\frac{{\sum }_{i=1}^{n}\left({X}_{i}-\bar{X}\right)\left({Y}_{i}-\bar{Y}\right)}{\sqrt{{\sum }_{i=1}^{n}\left({X}_{i}-\bar{X}\right)}\sqrt{{\sum }_{i=1}^{n}\left({Y}_{i}-\bar{Y}\right)}}\right]}^{2}$$

6 $$nse=1-\frac{{\sum }_{i=1}^{n}{\left({X}_{i}-{Y}_{i}\right)}^{2}}{{\sum }_{i=1}^{n}{\left({X}_{i}-\bar{X}\right)}^{2}}$$

7 $$d=1-\frac{{\sum }_{i=1}^{n}\left|{Y}_{i}-{X}_{i}\right|}{{\sum }_{i=1}^{n}\left(\left|{Y}_{i}-\bar{X}\right|+\left|{X}_{i}-\bar{X}\right|\right)}$$

Where, *X*_*i*_ and *Y*_*i*_ are the ith observations of datasets *X* and *Y*, $\bar{X}$ and $\bar{Y}$ are the means of datasets X and Y, and *n* is the number of the observations.

We wanted as minimal change as possible in the interferogram data in order to be able to compare it to the effect that may be produced by one approach or another. This is done to assess if the correction increases final result uncertainty. Therefore, we selected interferograms dates which do not exhibit deformation.

## Data Records

ADTC-InSAR^53^ are data sets containing the average daily tropospheric delays corresponding to the day of each year for 10 volcanoes in the Andes: Nevados del Ruiz, Galeras, Reventador, Hualca-hualca, Uturuncu, Robledo, Copahue, Llaima, Cordón Caulle and Chaitén. The database provides the average daily tropospheric delay for the total as well as its wet and dry components. For each of them, and for every month, one file is provided per volcano (36 files for each volcano). These files each include columns containing the average daily tropospheric delays for each day of the relevant month. In addition, the longitude and latitude files of the points for each volcano where the average daily tropospheric delays were measured are supplied.

Also, for the user to employ ADTC-InSAR as a tropospheric correction, the Corr__ADTC_InSAR.m and Corr__ADTC_InSAR.py scripts, executable in MATLAB and PYTHON, respectively, are also provided.

Finally, a README file providing this detailed information is also included.

## Technical Validation

This section presents two approaches for assessing the usefulness of ADTC-InSAR as a database for tropospheric corrections in InSAR data: (1) climate-related behavior of tropospheric delay and its components, and (2) ADTC-InSAR application to interferograms.

### Climate-related behavior of daily tropospheric delay and its components

**Fig. 2:**
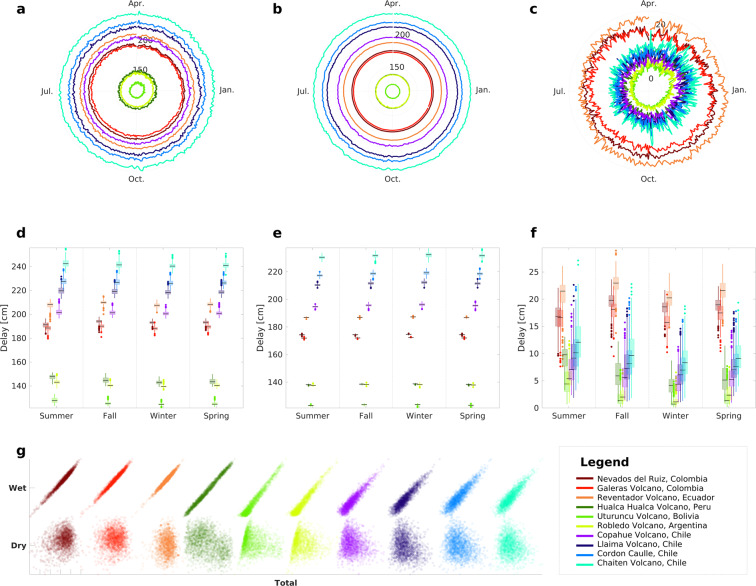
Comparison between total, dry and wet average daily tropospheric delay: (**a**–**c**) Polar plots illustrating the mean of average daily tropospheric delay of the (**a**) total, (**b**) dry, and (**c**) wet daily delay for each volcano, with the months progressing clockwise and the delays’ magnitudes labeled on the radial axis. (**d**–**f**) The graphs depict boxplots of the (**d**) total, (**e**) dry, and (**f**) wet daily tropospheric delay for all volcanoes during each season of the year (summer, fall, winter, spring) corresponding to the southern hemisphere. (**g**) The figure depicts scatter plots between the daily total tropospheric delays and their wet (top) and dry (bottom) components for each volcano. Each volcanic zone is distingished by a spectrum of different colors: Northern Volcanic Zone is a range of red colors (Brown: Nevados del Ruiz, red: Galeras, orange: Reventador), Central Volcanic Zone is range of green colors (dark green: Hualca hualca, light green: Uturuncu, lemon green: Robledo), and Southern Volcanic Zone is a range of blue colors (lilac: Copahue, purple: Llaima, blue: Cordón Caulle, cyan: Chaitén).

Examining the temporal dynamics of a daily tropospheric delay requires first identifying whether its components rise, decrease, or remain constant over the year. We also attempt to understand better why these changes occur and the connection between volcanic zones. Due to the cyclical nature of the process, the average daily tropospheric delay is shown on polar graphs (Fig. 2a–c). Boxplots and histograms illustrate the seasonal variation of extreme values (Fig. 2d–f). Second, the link between wet, dry, and total tropospheric delay is then shown using scatter plots (Fig. 2g). In addition, the percentage contribution of the wet and dry component to the total are estimated (Table 2). This enables us to deduce which component of the daily tropospheric delay substantially impacts the total amount and whether differences are detected between volcanic zones.

**Table 2:** Table displaying the percentage contribution of the dry (third column) and wet (fourth column) daily delays to the total daily lag of each volcano (second column) in the volcanic zones (first column) studied.

Volcanic zone	Volcano	Dry [%]	Wet [%]
NVZ	Nevados Del Ruiz	90.3	9.7
	Galeras	91.0	9.0
	Reventador	89.6	10.4
CVZ	Hualca-hualca	96.0	4.0
	Uturuncu	98.8	1.2
	Robledo	98.4	1.6
SVZ	Copahue	97.3	2.7
	Llaima	96.7	3.3
	Cordón Caulle	96.4	3.6
	Chaitén	96.0	4.0

According to what is observed in the Koppen-Geiger climate data (see Fig. 1, and also Table 2 in Beck *et al*.^15^), NVZ has a substantial tropical climates presence in all the variables (Af, Am, and Aw). It is characterized by high temperatures (T° > 18[°C] in the coldest months) and copious precipitation (≥60 [mm/month] in the driest months). All these characteristics indicate a high humidity level. Type Cfb, which lacks dry seasons but has hot summers (>10[°C]), is the second most-prevalent type of temperate climate type. In the warmer months, Polar-tundra (ET) type temperatures range between 0–10[°C]. As shown in Fig. 1, precipitations vary significantly throughout the year. Compared to other volcanic zones (CVZ and SVZ), the temperature at the height of 2 meters is relatively high and stable. Roncancio *et al*.^54^ describe these zones as having a temperate, cool-to-cold, or extremely cold climate, with lowest and maximum daily temperatures, respectively, below 15 °C and 25 °C. The temperature behavior is significantly influenced by the orogenic characteristics of the Andes, humidity, and winds from the low plains, all of which influence the bimodal rainy regime^54^.

CVZ climates correlate to arid zones characterized by desert and steppe climates (BWk and BSk), with monthly temperatures below 18[°C]. The polar tundra climate (ET) is caused by the high elevation temperature of the Andes^55^, with temperatures ranging between −5 and 10[°C] (Fig. 1). In this geographical area, the so-called Altiplano winter depicted in Fig. 1 is present, which is accompanied by summer rains. These are the result of local fluctuations in solar insolation and are closely linked to changes in large-scale circulation, such as variations in the supply of moisture east of the central Andes^56^.

According to Fig. 1, the prevailing climate in the SVZ corresponds to a temperate climate, mostly due to the decrease in elevation of the Andes, the impact of the westerly winds, the high precipitation, and the oceanic conditions^55^. It depicts a hot summer with a dry season or without a dry season at all (Csb or Cfb, respectively). Consequently, the average temperature throughout the warmest months is regularly over 10[°C]. In contrast, the average temperature during the coldest months ranges from 0 (or even lower) to 18[°C]. Figure 1 illustrates that precipitations vary significantly throughout the year, whereas seasonal temperatures range between −5° to 15 °C. Having described and recognized that the predominant climates in NVZ, CVZ, and SVZ are tropical, desert, and temperate, respectively, it is conceivable to evaluate the ADTC-InSAR database obtained in the volcanoes of this study (see Fig. 1).

When considering volcanic climatic zones, the behavior of daily tropospheric delay is better understood. Following are some distinguishing features of NVZ, CVZ, and SVZ. NVZ, for instance, has a tropical rainforest climate (Fig. 1), which means a high humidity percentage. Consequently, it is easy to see why the NVZ region has greater wet daily tropospheric delays than CVZ and SVZ regions (see Fig. 2c). Due to the NVZ’s location on the equator, this region exhibits distinct climatic behaviors: bimodality (two dry and two wet seasons) and unimodality (one dry and one wet season)^57^. Indeed, the region’s central-south wet seasons begin in February-March (summer) and September-October (spring). The dry periods occur in June (winter) and December (summer). These dates coincide with periods (between spring and autumn) with increased tropospheric wet delay in the NVZ volcanoes (Fig. 2f). Thus, it increases from a mean value of 18.07 ± 1.64, and 17.94 ± 3.46 [cm] in winter and summer (mainly dry periods) to 19.21 ± 1.79 [cm], and 20.15 ± 1.74 [cm] on average in spring and autumn (mainly wet seasons), respectively. On the other hand, the large dry tropospheric delay magnitudes (Fig. 2b,e) had mean values of 177 ± 0.21 [cm], which might be a result of high temperatures since the dry component is a function of atmospheric temperature and pressure^42,43^.

The CVZ volcanoes, Uturuncu and Robledo, have an arid-cold-desert climate (Fig. 1). Figure 1 depicts the polar-tundra climate of the Hualca-hualca volcano (also in CVZ), which implies low temperatures and low humidity. These climatic conditions may explain the lower magnitudes of the daily wet and dry tropospheric delays compared to other zones (see Fig. 2b,c,e,f). Notably, the wet daily tropospheric delay rises in the CVZ volcanoes from December to April (summer-autumn, see Fig. 2c). In the spring-summer seasons, there is also an increase in the seasonal data distribution (Fig. 2f). The latter may have something to do with the so-called Altiplanic winter. Changes in the intensity and duration of the Altiplano humid airflow’s intensity and duration^58^ enhance precipitation during summer, between December and February^59^.

The high dry daily tropospheric delay values in the SVZ are consistent with a temperate climate with warm summers. However, because temperatures in this zone are lower than in tropical zones such as NVZ, these delays are not as large. Likewise, precipitation is greater in NVZ, resulting in shorter wet daily tropospheric delays in SVZ.

Misra and Enge^10^ reported that the wet component accounts for just 10% of the total delay. The total contribution of the wet component in the NVZ is ten percent, as seen in Table 2. In contrast, this proportion falls to less than 5% in all other locations (CVZ and SVZ). The drop in the humid contribution results from the lower humidity and temperature variations in this area since the temperature at which a change in humidity is triggered can considerably impact the resulting delay^60^. Thus, outstanding contributions to the total daily tropospheric delay are restricted to tropical climate zones. Regardless, the dry contribution is much more significant than the wet contribution. It controls the total daily delay magnitude.

As demonstrated in Fig. 2g, correlations between the wet-to-total daily tropospheric delay, are more than 90% in all regions. Moreover, based on the positions of the extreme values (Fig. 2a–c), the distributions of wet and total daily tropospheric delays are equivalent. It demonstrates that the total daily tropospheric delay variations may be conditioned by its humid contribution.

In conclusion, we determined that it is essential to establish the tropospheric correction depending on the position of a volcano in a specific climate condition. We also noted that despite the substantial difference between dry and wet contributions, the wet contribution dominates the correction with “specific-dates”: the dry contribution varies less with time, whereas the wet part varies considerably. The variability in relative humidity has a considerable impact on the wet component of the daily tropospheric delay. As a result, the wet contribution would be associated with the rainy seasons of these zones, such as the Altiplanic winter in CVZ and the wet seasons in NVZ. Consequently, a more thorough assessment of relative humidity derived from satellite missions or *in-situ* meteorological data may improve the local tropospheric correction for the InSAR data.

### Application of ADTC-InSAR to interferograms

We assess the pertinence of ADTC-InSAR by statistically comparing its findings to “TRAIN specific-dates”. It was accomplished in two ways: first, by generating corrections for 500 randomly chosen date pairs at each volcano, and second, by applying the method to real interferograms.

**Fig. 3:**
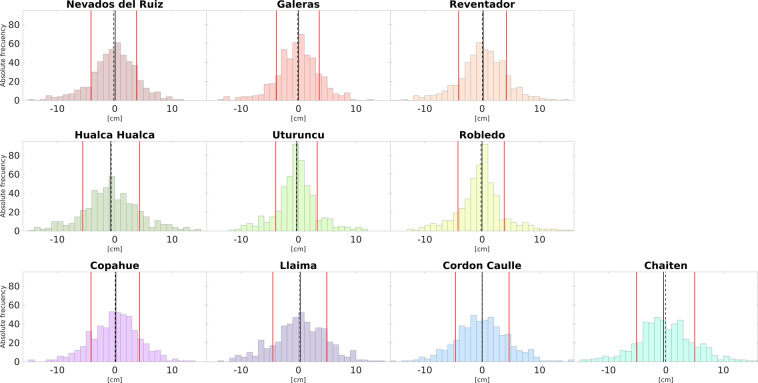
Histogram plots illustrate differences in tropospheric corrections between the “TRAIN specific-dates” and the ADTC-InSAR for the ten volcanoes in this study using 500 pairs of random dates. The histograms depicts three statistical parameters: the mean (solid black line), the median (segmented black line), and the standard deviation range (solid red line).

The difference between “TRAIN specific-dates” and ADTC-InSAR corrections for 500 random date pairings is calculated and illustrated using histograms (see Fig. 3), while the statistics for these data distributions are included in Table 3.

**Table 3:** The table presents different statistical indicators for each data set of the difference between applying tropospheric corrections with “TRAIN specific-dates” and ADTC-InSAR in 500 pairs of different dates in each volcano (Fig. 3).

	Nevados Del Ruiz	Galeras	Reventador	Hualca-hualca	Uturuncu	Robledo	Copahue	Llaima	Cordón Caulle	Chaitén
Median [cm]	0.07	0.01	0.17	−0.70	−0.28	−0.03	0.20	0.31	−0.02	−0.42
Mean [cm]	−0.17	−0.12	0.05	−0.62	−0.35	−0.20	0.09	0.24	0.01	−0.05
Standard deviation [cm]	3.97	3.74	4.16	4.96	3.61	4.03	4.23	4.70	4.67	5.06
*Q*_1_ [cm]	−2.27	−2.30	−2.09	−3.54	−2.11	−2.07	−2.53	−2.61	−2.98	−3.14
*Q*_3_ [cm]	2.34	2.22	2.66	2.21	1.55	1.59	2.71	3.45	2.85	2.89
Kurtosis	3.74	4.06	4.30	3.46	4.29	4.81	3.39	3.09	3.63	3.54
Skewness	−0.36	−0.32	−0.35	0.12	0.05	0.26	−0.14	−0.10	0.16	0.14

The statistics used are: the mean and median central tendency measurements, the standard variation, the firth and third quartiles (*Q*_1_ and *Q*_3_: accumulation of 25% and 75% of the data, respectively), kurtosis and skewness.

According to the statistics, the mean and median had comparable values ranging from −0.62 to 0.24, and −0.70 to 0.31 [cm], respectively, indicating that over the 500 cases, the extremes do not appear to have a significant impact in the mean, which are mainly centered about 0 [cm]. The leptokurtic kurtosis (K > 3) implies a larger accumulation of data near the mean and a rapid fall towards the extremes, whereas a skewness close to 0 suggests a symmetrical distribution. In addition, based on the values of quartiles (which contain 50% of the data), which vary from −2.41 to 2.50, and the standard deviation (red lines in Fig. 3), most of the discrepancies between the two databases lie between relatively small values. Based on that, we can infer that ADTC-InSAR database is, therefore a viable choice for generating InSAR data corrections.

However, the ADTC-InSAR database as being an average over several years may tend to minimize extremes in atmospheric effects. Consequently, there may be greater or lesser differences between the two correction databases depending on the degree of day-to-day variation at a particular location for a particular day. For instance, in the case of the Altiplano winter in the ZVC, this correlates to pairings of dates that contain days that are in periods with greater precipitation.

**Fig. 4:**
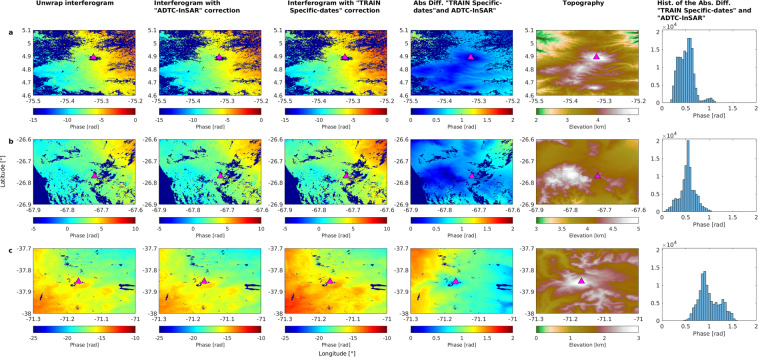
Application of tropospheric correction to a real interferogram at the (**a**) Nevados del Ruiz, (**b**) Robledo and (**c**) Copahue volcanoes: (first column) unwrapped interferograms, in Nevados Del Ruiz between 2008/03/06 and 2010/03/12 (path 447- frame 3520), in Robledo between 2010/03/18 and 2010/12/19 (path 101 - frame 6640), in Copahue between 2010/12/27 and 2011/02/11 (path 115 - frame 6410; see Table 1); (second column) unwrapped interferogram corrected with ADTC-InSAR; (third column) unwrapped interferogram corrected with “TRAIN specific-dates”; (fourth column) the absolute difference between the correction with ADTC-InSAR and with “TRAIN specific-dates”; (fifth column) the volcanoes topography in kilometers; and (sixth column) a histogram of the absolute difference shown on fourth colum. The X-axis of each graphs represent longitude, the Y-axis represents latitude, and the color bars represent phase in radians.

As stated previously, a comparison of corrections applied to interferograms of representative volcanoes from the three climatic zones was performed. Figure 4 shows ALOS-PALSAR data of Nevados del Ruiz (NVZ), Robledo (CVZ) and Copahue (SVZ) volcanoes. These unwrapped interferograms show no deformations, consistent with previous research. In detail, no studies indicate deformation in Nevados del Ruiz between the interferogram dates (March 06, 2008, to March 12, 2010). InSAR and GPS stations have detected deformations at this volcano^61,62^, an increase in seismic activity^63,64^, and the extrusion of a dome since 2015^65^. Even Reath *et al*.^66^ indicate that the volcano has behaved like an open system between approximately 2005 and 2012, with no magma injection or ejection from the reservoir. Since 1992, small deformations have been reported at the Robledo volcano^67–69^. Even between 2005 and 2010, deformations of 0.87 [cm/yr] were reported^69^, which are not visible in the interferograms of Fig. 4, because they do not cover the same period of time (March 18, 2010 and December 19, 2010) and their low magnitude. Lastly, deflations were detected in the Copahue volcano between 2002 to 2007^11,19,70^ and inflation has been observed since 2011^20,22^. However, the interferogram does not span the time period between these two events as it only considers from December 27, 2010, to February 11, 2011.

The second purpose is also to examine the spatial disparities these two correction databases can cause on the interferograms. As a result, “TRAIN specific-dates” and ADTC-InSAR tropospheric corrections were applied to all interferograms (Table 1). The estimation of the difference between the two approaches can be observed in Fig. 4, which displays the results for Nevados del Ruiz, Robledo and Copahue volcanoes. Observed in the first column are unwrapped interferograms. ADTC-InSAR and “TRAIN specific-dates” corrected interferograms are shown in the second and third columns. The fourth column shows the difference between both corrections (in absolute values). In column 5 is the topography of the area. In the sixth column is the difference’s histogram (column 4).

Table 4 presents the statistics pertaining to the comparison of both corrections applied to the interferograms (second and third columns in Fig. 4), followed by its difference (column 4 in Fig. 4) and the statistics of the histogram of column 6 (in Fig. 4). First, note that the spatial variability of the corrected interferograms (second and third column of Fig. 4) are spatially very similar. This is evidenced by the spatial standard deviations of the fields that do not differ significantly. Thus, the standard variations of the second column (corrected with ADTC-InSAR) are 1.71, 9.03 and 1.21[rad], and for the third column (corrected with “TRAIN specific-dates”) are 1.79, 8.82 and 1.32 [rad], for Nevados del Ruiz, Robledo, and Copahue, respectively. In the fourth column is the absolute spatial difference. In the case of Nevados del Ruiz and Robledo the difference between both corrections is centered at 0 [rad] (blue tones), while Copahue has mostly values closest to 1 [rad] (from yellow to cyan). It can also be noted that specifically over the summits (Nevados del Ruiz: [−75.32°, 4.89°]; Robledo: [−67.72°, −26.77°]; Copahue: [−71.17°, −37.85°]) is where the differences are the smallest. The latter is confirmed by the values of the histogram, which range from 0.19-to-1.18, 0.05-to-0.90, and 0.41-to-1.60 [rad] for Nevados del Ruiz, Robledo and Copahue, respectively, therein confirming the visual impression. Moreover, means and medians are comparable, with kurtosis varying between 2.39 and 3.34, confirming that extremes do not play a meaningful role. Instead, although skewness is low (<|0.5|), the difference of corrections established over 500 cases (Fig. 3) shows that, in general, the corrections are symmetrical. Therefore, the asymmetry for these dates is only a particular case.

**Table 4:** Statistical pertaining to corrections with “TRAIN specific-dates” and ADTC-InSAR. The median, mean, standard deviation, kurtosis and skewness statistics are applied to the difference between applying the “TRAIN specific-dates” correction and ADTC-InSAR to real interferograms (fourth column of Fig. 4), and at 500 cases of differences in corrections (Fig. 3).

		Nevados Del Ruiz	Robledo	Copahue
Difference between correction with “TRAIN specific-dates” and ADTC-InSAR	Median [rad]	0.50	0.54	0.94
	Mean [rad]	0.50	0.53	1.00
	Standard deviation [rad]	0.15	0.16	0.22
	Kurtosis	3.34	2.82	2.39
	Skewness	0.39	−0.47	0.43
**Comparison between correction with “TRAIN specific-dates” and ADTC-InSAR**	R^2^	0.9946	0.9862	0.9771
	nse	0.92	0.78	0.40
	d	0.83	0.73	0.57

*R*^2^, *nse* and *d* are utilized to compare “TRAIN specific-dates” and ADTC-InSAR corrections to real interferograms (second and third column of Fig. 4).

Lastly, spatial efficiency statistics between the two corrected fields (second and third columns of Fig. 4) are computed (see Table 4). First, spatial correlation is very high (*R*^2^ > 0.95) for all the volcanoes. Two efficiency metrics, the *nse* and the modified *d*, were also determined. These vary depending on the analyzed volcanoes and indicate that the ADTC-InSAR correction is very convenient to use in the Nevados del Ruiz (*nse* = 0.92 and d = 0.83) and Robledo (*nse* = 0.78 and *d* = 0.73) for these dates. Instead, the efficacy for the Copahue volcano, for these dates, is lower, with *nse* = 0.40 and *d* = 0.57. Despite the latter represents low efficiency values, they however imply that the corrections performed to the interferograms are in good agreement.

This approach allow concluding that both tropospheric correction procedures behave similarly with small differences on interferograms, most likely due to circumstances on the day the SAR measurements were taken, which are not discernible when using an average mesh, such as ADTC-InSAR.

## Usage Notes

ADTC-InSAR provides information on the total average daily tropospheric delay and its wet and dry components for each days of the year. It can be used to analyze tropospheric delay behavior and correct InSAR data for tropospheric effects. Nevertheless, it must be kept in mind that, depending on the volcano and climatic zone, there are times of the year when the wet component of the average daily tropospheric delay tends to rise (see Fig. 2a,b). Since these grids are average conditions, they may reduce the severity of unusual extreme precipitation circumstances, resulting in discrepancies with a correction based on “TRAIN specific-dates”.

The complete database weighs 49 GB, because ADTC-InSAR is a set of arrays within ASCII-files structures. As a result, the computational capabilities available must be considered. Each file [Abbr.]_era5_clim_[month] contains the average daily tropospheric delays (in cm) for each volcano, where [Abbr.] indicates the volcano according to Table 1, and [month] corresponds to the month. Each of these ASCII files contains a matrix of NxM, where each column contains the average daily tropospheric delays for the day of the month (M is the maximum number of days each month). It should be noted that each value n (<N) of each column has the longitude and latitude position contained in the [Abbr.]_ll files, respectively (ASCII-files containing 2-column arrays: longitude and latitude; these are found in the LONLAT_ASCII folder).

Finally, we intend to indicate why this database benefits the scientific community.

Our approach was centered on extreme events requiring effectiveness. Interferogram correction is laborious, time-consuming, and consequently often unattainable in emergency situations. Downloading atmospheric data, processing them with software, and addressing other obstacles requires professional software management, which is not within usual staff capabilities, especially in a crisis. This easily accessible database saves time in generating a preliminary estimate of what is occurring prior to the release of actual datasets, which is crucial for good emergency response.

In addition, a comprehensive description of the mean variability of tropospheric corrections over some specific volcanoes is valuable regardless of the occurrence of an extreme event. Practically, this database may be used to estimate the optimal time of year to gather InSAR data based on the behavior of the tropospheric delay throughout the year. It can already serve as a comparative reference for reanalysis purposes. Thus, in the future, in addition to expanding the temporal range covered by the daily means, so that they may be used as a reference for certain decades, we will expand the database geographically to cover not only additional volcanic zones, but also specific regions of interest for SAR research^71^.

We emphasize that, only a basic installation of Matlab or Python is required. This is convenient since it accommodates the user’s preferences and demands without requiring much effort.

Finally, based on all the above, this data set can also be used as a practical resource for undergraduate and postgraduate courses in geoscience institutions.

## Data Availability

ADTC-InSAR^53^ has been developed in MathWorks MATLAB version R2019a^72^ using TRAIN MATLAB software. It is possible to generate tropospheric corrections for the previously mentioned volcanoes using the Corr__ADTC_InSAR.m and Corr__ADTC_InSAR.py scripts.
